# Synergistic effects of baicalein with cefotaxime against *Klebsiella pneumoniae* through inhibiting CTX-M-1 gene expression

**DOI:** 10.1186/s12866-016-0797-1

**Published:** 2016-08-08

**Authors:** Wenhui Cai, Yingmei Fu, Wenli Zhang, Xiaobei Chen, Jizi Zhao, Wuqi Song, Yujun Li, Ying Huang, Zheng Wu, Rui Sun, Chunping Dong, Fengmin Zhang

**Affiliations:** 1 Wu Lien-Teh Institute, Department of Microbiology, Harbin Medical University, 157, Baojian Road, Nangang District, Harbin, 150081 China; 2 Department of Microbiology and Immunology, School of Basic Medical Sciences, Heilongjiang University of Chinese Medicine, Harbin, China; 3 Heilongjiang Provincial Key Laboratory of Infection and Immunity, Harbin Medical University, Harbin, China

**Keywords:** Baicalein, Extended- spectrum β- lactamases, *Klebsiella pneumoniae*, Synergistic antibacterial action, CTX-M-1 gene

## Abstract

**Background:**

Generation of extended- spectrum β- lactamases is one of the major mechanisms by which clinical *Klebsiella pneumoniae* develop resistance to antibiotics. Combined antibiotics prove to be a relatively effective method of controlling such resistant strains. Some of Chinese herbal active ingredients are known to have synergistic antibacterial effects. This study is aimed to investigate synergistic effects of Chinese herbal active ingredients with cefotaxime on the extended- spectrum β- lactamase positive strains of *Klebsiella pneumoniae*, and to analyze mechanism of synergistic action, providing experimental evidence for clinical application of antimicrobial drugs.

**Results:**

For total sixteen strains including fifteen strains of cefotaxime resistant *K. pneumoniae* and one extended- spectrum β- lactamase positive standard strain, the synergy rates of cefotaxime with baicalein, matrine, and clavulanic acid were 56.3 %, 0 %, and 100 %, respectively. The fractional inhibitory concentration index of combined baicalein and cefotaxime was correlated with the percentage decrease of cefotaxime MIC of all the strains (*r* = −0.78, *p* <0.01). In the group of synergy baicalein and cefotaxime, the transcribed mRNA level of CTX-M-1 after treatment of baicalein was decreased significantly (*p* <0.05). Moreover, the CTX-M-1 mRNA expression percentage inhibition (100 %, 5/5) was significantly higher than non- synergy group (25 %, 1/4) (*p* <0.05).

**Conclusions:**

Our study demonstrated that baicalein exhibited synergistic activity when combined with cefotaxime against some of extended- spectrum β- lactamases positive *K. pneumoniae* strains by inhibiting CTX-M-1 mRNA expression. However, no direct bactericidal or bacteriostatic activity was involved in the synergistic action. Baicalein seems to be a promising novel effective synergistic antimicrobial agent.

**Electronic supplementary material:**

The online version of this article (doi:10.1186/s12866-016-0797-1) contains supplementary material, which is available to authorized users.

## Background

Extended- spectrum β- lactamases (ESBLs) have the ability of hydrolyzing a variety of antibiotics, such as penicillin, cephalosporins, and monobactams. It is the main mechanism for the formation of various kinds of bacterial resistance. ESBL can be suppressed by commonly used β- lactamase inhibitors, such as clavulanic acid by binding to and inhibiting the activity of ESBL when combined with antibiotics. Combined antibiotics prove to be a relatively effective method of controlling such resistant strains [1]. However, in recent years, the emergence of resistant strains of β- lactamase inhibitors results in failure of interactive antibiotic treatment. Seeking for new and effective synergistic antimicrobial agents to overcome bacterial resistance are urgently needed.

Chinese medical herbs have been a rich resource for the discoveries of alternative synergistic antimicrobial agents. Several studies show that certain active ingredients of Chinese herbs have synergistic inhibitory effects on bacteria with antibiotics, such as baicalein and matrine [2, 3]. Baicalein is a type of flavonoids from the roots of *Scutellaria baicalensis* and *Scutellaria lateriflora*, which is one of the most commonly used Chinese herbs in China for the treatment of bacterial infections [4]. Synergies of baicalein were identified in combination with tetracycline or β-lactams against two methicillin-resistant *Staphylococcus aureus* (MRSA) clinical isolates OM481 and OM584 [2]. Baicalein was also reported to have synergy with gentamicin against vancomycin-resistant *Enterococcus* [5]. Chan et al. reported synergistic effects of baicalein with ciprofloxacin against NorA over-expressed methicillin-resistant MRSA [6].

*Klebsiella pneumoniae* (*K. pneumoniae,* KP) is a type of Gram-negative bacteria that can cause different types of infections, including pneumonia, bloodstream infections, wound or surgical site infections, and meningitis (http://www.cdc.gov/HAI/organisms/klebsiella/klebsiella.html). Increasingly, *Klebsiella* bacteria have developed antimicrobial resistance with a higher detection rate of ESBL [7, 8]. With a wide range of therapeutic benefits, the synergy of baicalein with other antibiotics against *K. pneumoniae* may be identified. The aim of the present study was to investigate antibacterial effects of baicalein in association with cefotaxime against ESBL positive *K. pneumoniae* compared with another candidate Chinese herbal ingredient named matrine, which is a kind of alkaloids containing lactam ring structure from the *Sophora* genus. Moreover, possible mechanisms by which baicalein interacts with cefotaxime against *K. pneumoniae* were studied.

## Methods

### Reagents and Chinese herbal active compounds

Cefotaxime was purchased from Harbin Pharmaceutical Group Co., LTD General Pharm Factory. Clavulanic acid and baicalein were purchased from Sigma. Matrine was purchased from National Institutes for Food and Drug Control. Cefotaxime and matrine were dissolved in sterile water. Baicalein was dissolved in dimethyl sulfoxide (DMSO) whose final concentration was less than 1 % according to the Clinical and Laboratory Standards Institute (CLSI, USA). Clavulanic acid was dissolved in Phosphate buffer (pH 6.0, 0.1 mol/L).

### Collection of ESBL positive *K. pneumoniae* clinical isolates and identification

The clinical isolate**s** of ESBL positive *K. pneumoniae* were collected in the Affiliated Hospital of Harbin Medical University. They were identified using an API20E system (bioM´erieux, Marcy I’Etoile, France) with conventional biochemical methods. Finally 15 strains were randomly selected for this experiment. Quality control strain *Escherichia coli* ATCC 25922 and *K. pneumoniae* ATCC 700603 were kept in our laboratory.

### Measurement of β- Lactamase activity of clinical isolates of *K. pneumoniae*

β- Lactamase activity was assessed by nitrocefin test. The ESBL- producing strains were validated according to CLSI recommended methodology [9].

### Determination of the minimum inhibitory concentration (MIC)

MIC was defined as the lowest concentration of a drug that prevents visible growth of a bacterium. All drugs were diluted in Mueller-Hinton Broth (MHB). Each test well contained bacteria in a final concentration of 5 × 10^5^ CFU/mL. After 17 h’ incubation at 37 °C, they were checked for growth. *Escherichia coli* ATCC 25922 was used as sensitivity control strain. All experiments were repeated three times.

Since baicalein is colorful, we determined to use combined visual observation and spectrophotometer method to identify the MIC of drugs. The OD value of each well was read at 630 nm wavelength. The growth of bacteria after treatment was calculated using formula: bacterial growth rate = 100 % × OD_drug- containing well_/OD_drug- free well_, where OD value is obtained by subtracting the background OD value from the measured value in each well. MIC was determined as the lowest concentration of the drug on the inhibition rate of more than 90 % [6].

### Synergy testing of Chinese herbal active compounds with antibiotic using checkerboard dilution method

To investigate if baicalein and matrine have synergy with cefotaxime against *K. pneumoniae* in vitro*,* checkerboard dilution method was used [10]. Two drugs were diluted in MHB into 8 gradient concentrations, i.e., 1/32 × MIC- 4 × MIC, each longitudinal column of wells having the same concentration of drug A, and each horizontal row of wells having the same concentration of drug B. The total volume of each well was 200 μL, including 50 μL of drug A, 50 μL of drug B, and 100 μL of bacterial suspension with a final bacterial concentration of 5 × 10^5^ CFU/mL. In addition, single drug MIC control wells, drug- free control wells, bacteria- free control wells were established. *Escherichia coli* ATCC 25922 was used as sensitivity control strain. After incubation at 37 °C for 17 h, the MIC value was read. Each experiment was repeated three times. Synergy was determined by calculating the fractional inhibitory concentrations index (FICI) using formula: FICI = MIC _drug A combined with_/MIC _drug A used alone_ + MIC _drug B in combination with_/MIC _drug B alone_, where MIC _drug A combined with_ denotes the MIC of drug A when used in combination, MIC _drug A used alone_ denotes the MIC of drug A when used alone, MIC _drug B in combination with_ means the MIC of drug B when used in combination, and MIC _drug B alone_ means the MIC of drug B when used alone. Based on the FICI, the results of the interactive effects were as follows: FICI≤0.5 means synergy, 0.5 <FICI≤0.75 means partial synergy, 0.76 <FICI≤1 means additive, 1 <FICI≤4 denotes indifferent, FICI> 4 indicates antagonistic [10]. In this study, the synergy and the partial synergy were defined as synergy relationship, while the additive, the indifferent and the antagonistic were classified as non- synergy relationship, in order to facilitate statistical analysis.

### Detection of *bla*_SHV_, *bla*_TEM_, *bla*_CTX-M-1_, *bla*_CTX-M-9_ in clinical isolates of *K. pneumoniae*

Genomic DNA as templates were prepared using boiling pyrolysis method from clinical isolate**s** of *K. pneumoniae*. Specific PCR primers for genes *bla*_SHV_, *bla*_TEM_, *bla*_CTX-M-1_ and *bla*_CTX-M-9_ were determined in our previous study [9, 11] listed in Table 1. PCR reaction conditions were as follows: initial denaturation at 94 °C for 3 min, followed by 25 cycles of denaturation at 94 °C for 30 s, annealing for 30 s, and extension at 72 °C for 1 min, then extension at 72 °C for 5 min. PCR product was subjected to 1.2 % agarose gel electrophoresis, followed by staining and examination.

**Table 1:** Primers for ESBLs detection by PCR

Primer	Sequence(5’ → 3’)	Nuleotide position	Tm	Genbank accession No.	Size
SHV-F	TCTCCCTGTTAGCCACCCTG	224-243	59 °C	AF124984	593 bp
SHV-R	CCACTGCAGCAGCTGCCGTT	797-816			
TEM-F	GTATCCGCTCATGAGACAATA	154-174	56 °C	AB194682	717 bp
TEM-R	AGAAGTGGTCCTGCAACTTT	851-870			
CTX-M1-F	CGCTTTGCGATGTGCAG	264-280	56 °C	X92506	551 bp
CTX-M1-R	ACCGCGATATCGTTGGT	798-814			
CTX-M9-F	ATGGTGACAAAGAGAGTGCA	132-151	56 °C	AJ416345	868 bp
CTX-M9-R	CCCTTCGGCGATGATTCTC	983-1000			

### Measurement of mRNA transcriptional expression levels of *bla*_TEM_, *bla*_CTX-M-1_ and *bla*_CTX-M-9_ in the clinical isolates of *K. pneumoniae* by reverse transcription (RT)-PCR

Total RNAs were isolated using TRIzol (Invitrogen, Carlsbad, CA) method [9] from the bacteria. Random primers (Takara) and Moloney murine leukaemia virus reverse transcriptase (Promega) were used for RT, then PCR was run using bacterial 16SrRNA as internal control, primers 5' -GGA CGG GTG AGT AAT GTC- 3 'and 5' -ACA CCT GGA ATT CTA CCC- 3 '. The expected amplified fragment was 578 bp, and the annealing temperature was 56 °C. The primers and other reaction conditions were the same as in Table 1. The product was subjected to 1.2 % agarose gel electrophoresis. Then it was stained and analysis of target band was performed using grayscale analysis software Image J to generate relative mRNA expression levels, The intensity was expressed as a value relative to that of the 16SrRNA [12]. Each experiment was repeated three times.

### Counting of viable *K. pneumoniae* after treatment of baicalein and measurement of transcriptional expression of ESBL genes

To further understand the mechanisms by which baicalein works in combination with cefotaxime against these clinical isolates, we repeated the experiments with baicalein alone at the lowest inhibitory concentration determined during combination. Baicalein was added to the same MHB with clinical strains at the lowest inhibitory concentration determined when used in combination with cefotaxime. Bacterial concentration was 5 × 10^5^ CFU/mL. Blank control without baicalein was used for comparison. After 17 h’ incubation at 37 °C, 50 μL of bacterial suspension was taken for serial 10-fold dilution. Approximately 10 μL of bacterial inoculum was inoculated on the medium of agar plates for 17 h at 37 °C. Then viable bacterial counting was conducted. All tests were performed in triplicate. The results were expressed as mean ± standard deviation using CFU/mL as unit. At the same time, the mixed baicalein and bacterial inoculum was used for total RNA isolation. RT- PCR performed in the same ways as above. Each experiment was repeated three times.

### Statistical analysis

Statistical analysis was performed using the Fisher’s Exact Test, Student’s *t* test and correlation analysis with SPSS 16.0 software. *p* <0.05 was considered statistically significant.

## Results

### Interactive antibacterial effects of Chinese herbal active ingredients and clavulanic acid with cefotaxime

**Fig. 1:**
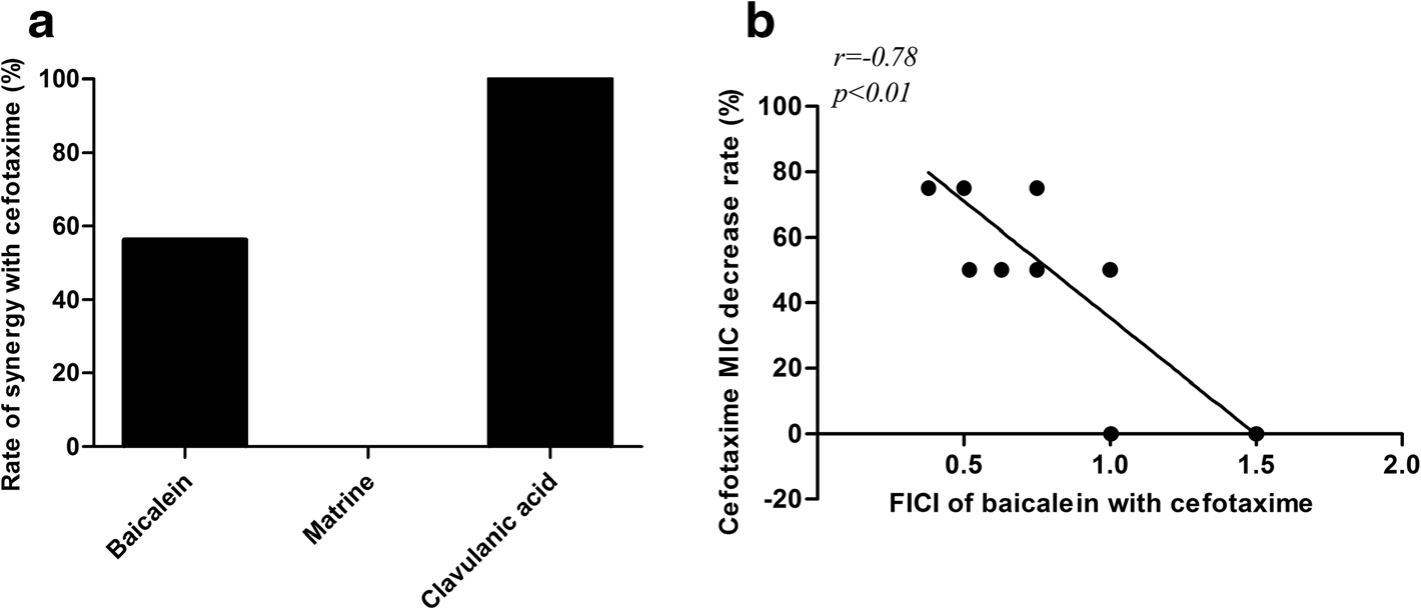
Synergy comparison and correlation analysis of FICI with cefotaxime MIC value decrease. (The synergy testing of baicalein, matrine, and clavulanic acid with cefotaxime in bacterial inhibition showed that different synergy rates, which is the percentage of synergistic strains among the total strains studied, were observed with cefotaxime (**a**). Correlation between the FICI of baicalein with cefotaxime and baicalein-induced cefotaxime MIC decrease percentage was analyzed using SPSS (**b**). *X-axis* denotes the FICI of baicalein with cefotaxime, *Y-axis* means cefotaxime MIC decrease percentage)

To investigate if baicalein can interact with cefotaxime in the control of *K. pneumoniae,* synergy testing was conducted on baicalein, matrine, and clavulanic acid with cefotaxime using checkerboard dilution method. The results (Table 2, Fig. 1a) showed that when combined with cefotaxime, baicalein exhibited synergistic effects on some antibiotic- resistant ESBL- positive strains of *K. pneumoniae* (56.3 %). But no synergy was observed with matrine (0 %). On the contrast, the positive control drug clavulanate acid showed 100 % synergistic. These findings indicated that baicalein may have moderate synergy with cefotaxime against *K. pneumoniae* in vitro*.* A further correlation analysis demonstrated that the FICI of baicalein and cefotaxime was negatively correlated with the percentage of cefotaxime MIC decrease (*r* = -0.78, *p* <0.01) (Fig. 1b).

**Table 2:** Interactive effects of Chinese herbal active ingredients with cefotaxime on antibiotic resistant *K. pneumoniae*

Strains No.	MIC_alone_(μg/mL)				MIC_combined_(μg/mL)		^a^FICI	MIC_combined_(μg/mL)		^a^FICI	MIC_combined_(μg/mL)		^a^FICI
	^b^Bai	^c^Mat	^d^Cla	Cefotaxime	^b^Bai	cefotaxime		^c^Mat	cefotaxime		^d^Cla	cefotaxime	
28	>256	>256	16	128	128	128	1.5	2	128	1.008	0.5	4	0.063*
30	>256	>256	8	128	64	64	0.75*	2	128	1.008	0.5	4	0.094*
58	>256	>256	32	256	128	128	1	2	256	1.008	0.5	8	0.047*
64	>256	>256	32	256	1	256	1.004	2	256	1.008	0.5	8	0.047*
80	>256	>256	32	512	64	256	0.75*	2	1024	2.008	0.5	16	0.047*
90	>256	>256	8	1024	128	1024	1.5	2	1024	1.008	0.5	32	0.094*
102	>256	>256	16	128	32	64	0.63*	2	128	1.008	0.5	4	0.063*
116	>256	>256	8	128	128	64	1	2	128	1.008	0.5	4	0.094*
171	>256	>256	16	128	128	64	1	2	128	1.008	0.5	4	0.063*
179	>256	>256	8	1024	64	256	0.5*	2	1024	1.008	0.5	32	0.094*
210	>256	>256	8	256	128	64	0.75*	2	256	1.008	0.5	8	0.094*
219	>256	>256	8	1024	1	1024	1.004	2	1024	1.008	0.5	32	0.094*
796	>256	>256	8	256	32	64	0.38*	2	256	1.008	0.5	8	0.094*
826	>256	>256	8	1024	128	256	0.75*	2	1024	1.008	0.5	32	0.094*
863	>256	>256	8	256	128	64	0.75*	2	256	1.008	0.5	8	0.094*
700603	>256	>256	8	4	4	2	0.52*	2	4	1.010	1	1	0.38*

*FICI ≤0.75 means synergy group including both synergy and partial synergy

^a^FICI≤0.5 synergy, 0.5 <FICI≤0.75 partial synergy, 0.76 <FICI≤1 additive, 1 <FICI≤4 indifferent, FICI> 4 antagonistic

^b^Bai:baicalein; ^c^Mat:matrine; ^d^Cla:clavulanate acid

### Number of *K. pneumoniae* after baicalein treatment in interactive concentrations

**Fig. 2:**
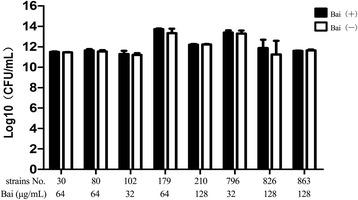
Effects of interactive concentration baicalein on the growth of *K. pneumoniae.* (For each strain, Bai (+) and Bai (−) were compared. Bai (+) denotes baicalein treated strain; Bai (−) denotes blank control strain. Each experiment was conducted in triplicate. *X-axis* denotes bacterial strain ID; *Y-axis* means log_10_ value of bacterial numbers)

To further investigate if baicalein can directly inhibit bacterial growth independently, the strains of clinical ESBL positive *K. pneumoniae* in synergy group was treated alone with baicalein at the same lowest inhibitory concentration determined when used in combination with cefotaxime. Each strain was treated both by baicalein alone and no baicalein. After incubation, counting of viable bacteria was conducted. The viable bacterial counting revealed that there was no significant difference (*P* > 0.05) in the number of viable bacterial colonies between baicalein treated and blank control groups (Fig. 2). This finding suggests that baicalein may not have direct bactericidal action when used in combination with cefotaxime against *K. pneumoniae.*

### Distribution of ESBL genes and their mRNA expression changes in *K. pneumoniae* treated with interactive concentration baicalein

**Fig. 3:**
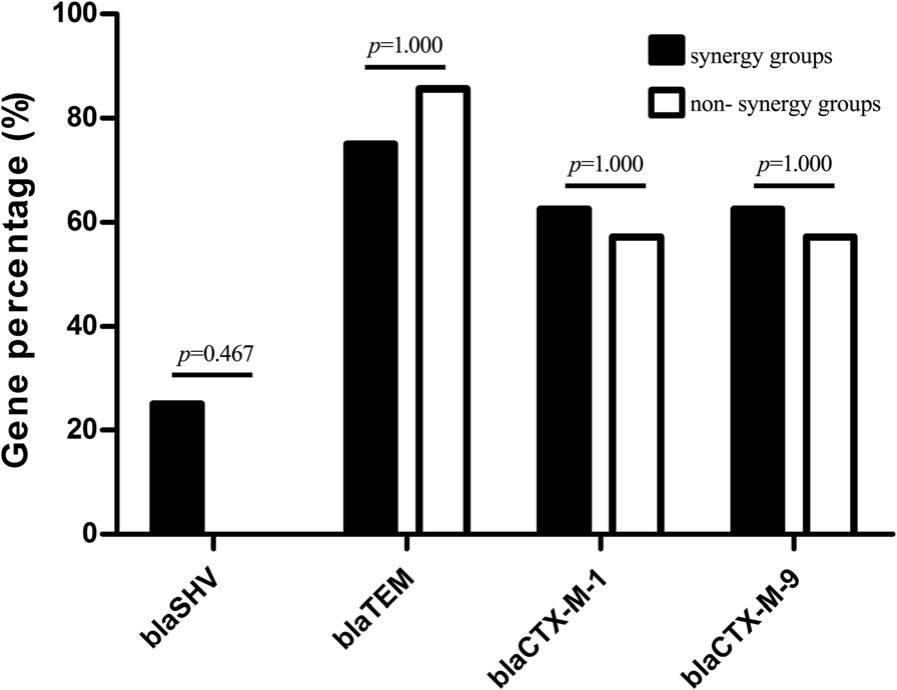
Comparison of ESBL gene percentage among different groups. (The percentage of four common ESBL resistance genes in the synergy group and non-synergy group was compared using Fisher’s Exact Test with SPSS software, *p* <0.05 was considered statistically significant. *Black columns* represent the percentage of the target genes in the synergy group, while *white columns* denote the percentage of the target genes in the non-synergy group)

To investigate if the synergy of baicalein with cefotaxime is associated the distribution of resistant genes in the clinical strains of *K. pneumoniae*, the percentages of ESBL resistant genes, including *bla*_SHV_、*bla*_TEM_、*bla*_CTX-M-1_、*bla*_CTX-M-9_ were compared between synergy group and non-synergy group (Fig. 3). The results showed that there were 2 strains with *bla*_SHV_ in the synergy group; 12 strains with *bla*_TEM_ both in synergy group and non-synergy group (each n = 6). The percentage of *bla*_TEM_ was 75 % in the synergy group and 85.7 % in non-synergy group. There were 9 strains with *bla*_CTX-M-1_, including 5 strains in synergy group with 62.5 % and 4 strains in non-synergy group with 57.1 %. There were 9 strains with *bla*_CTX-M-9_, including 5 strains in the synergy group with 62.5 % and 4 strains in the non-synergy group with 57.1 %. Comparison analysis showed that there was no significant difference in the distribution of the four common ESBL resistance genes (*P* > 0.05), suggesting that the synergy of baicalein and cefotaxime may not be associated with the distribution of these resistance genes.

**Fig. 4:**
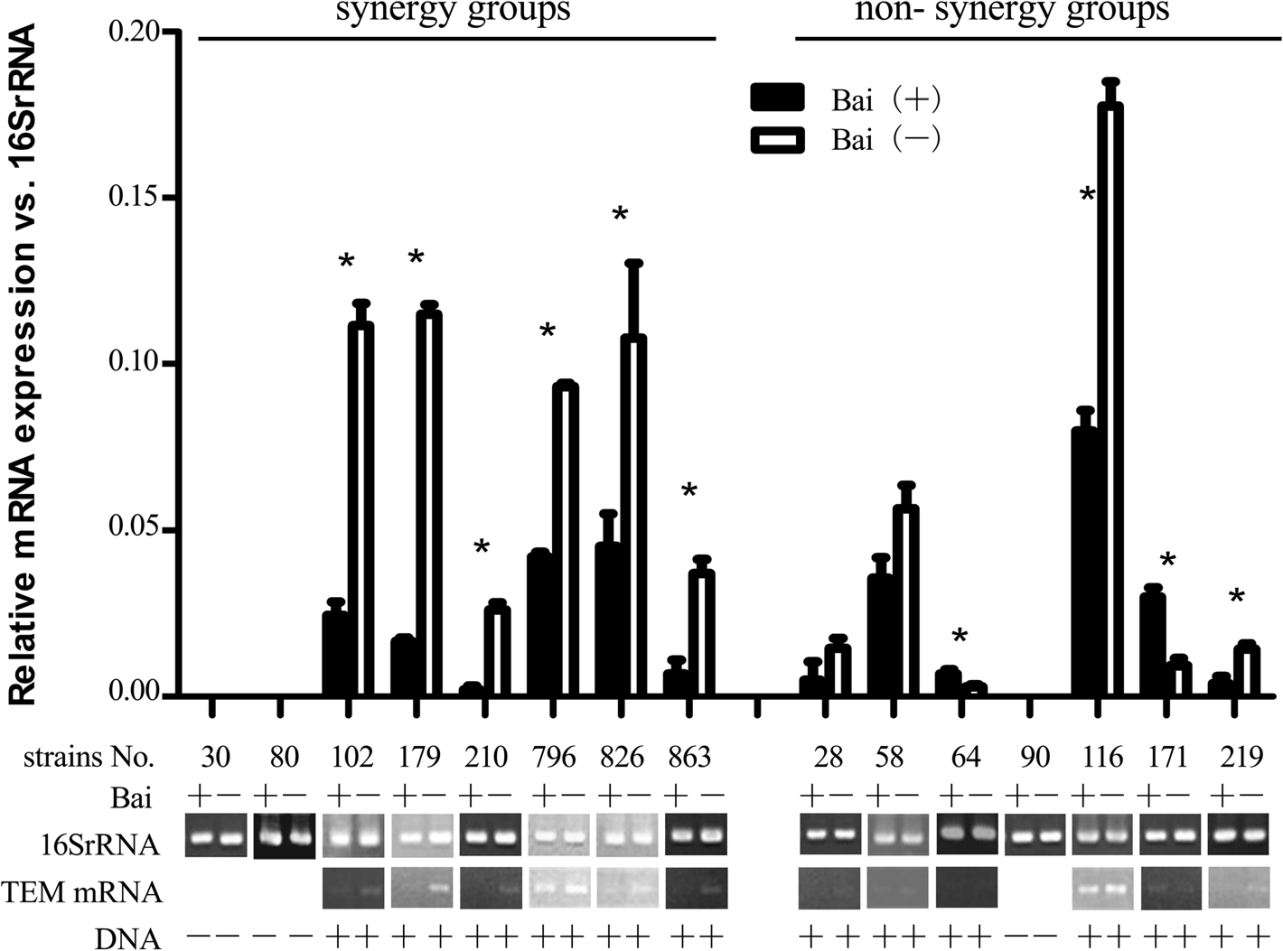
Effect of baicalein on the mRNA expression of TEM. (Bai (+) denotes baicalein treated strain in *black columns*; Bai (−) means blank control strain in *white columns*. Each strain was divided into baicalein treated and blank control subgroups for comparison of the effect of baicalein on mRNA expression. RT-PCR products were analyzed using Image J software. The mRNA level was expressed as the *gray* value of target gene relative to that of the 16SrRNA. Each experiment was done in triplicate. The mRNA value was expressed as mean ± standard deviation. The difference was analyzed using Student’s *t* test. *p < 0.05 meaning statistically significant)

**Fig. 5:**
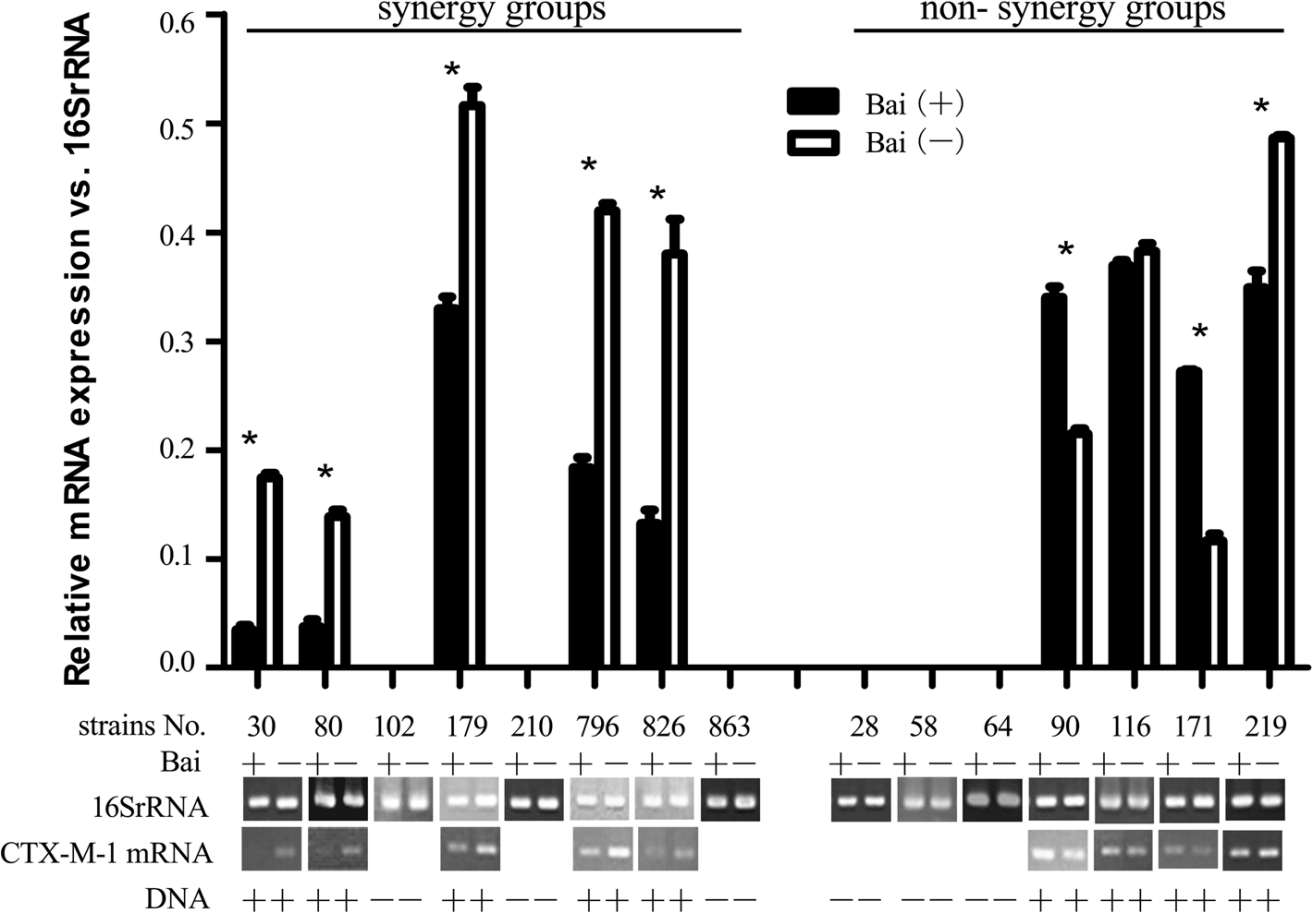
Effect of baicalein on the mRNA expression of CTX-M-1. (same as Fig. 4 in explanation)

**Fig. 6:**
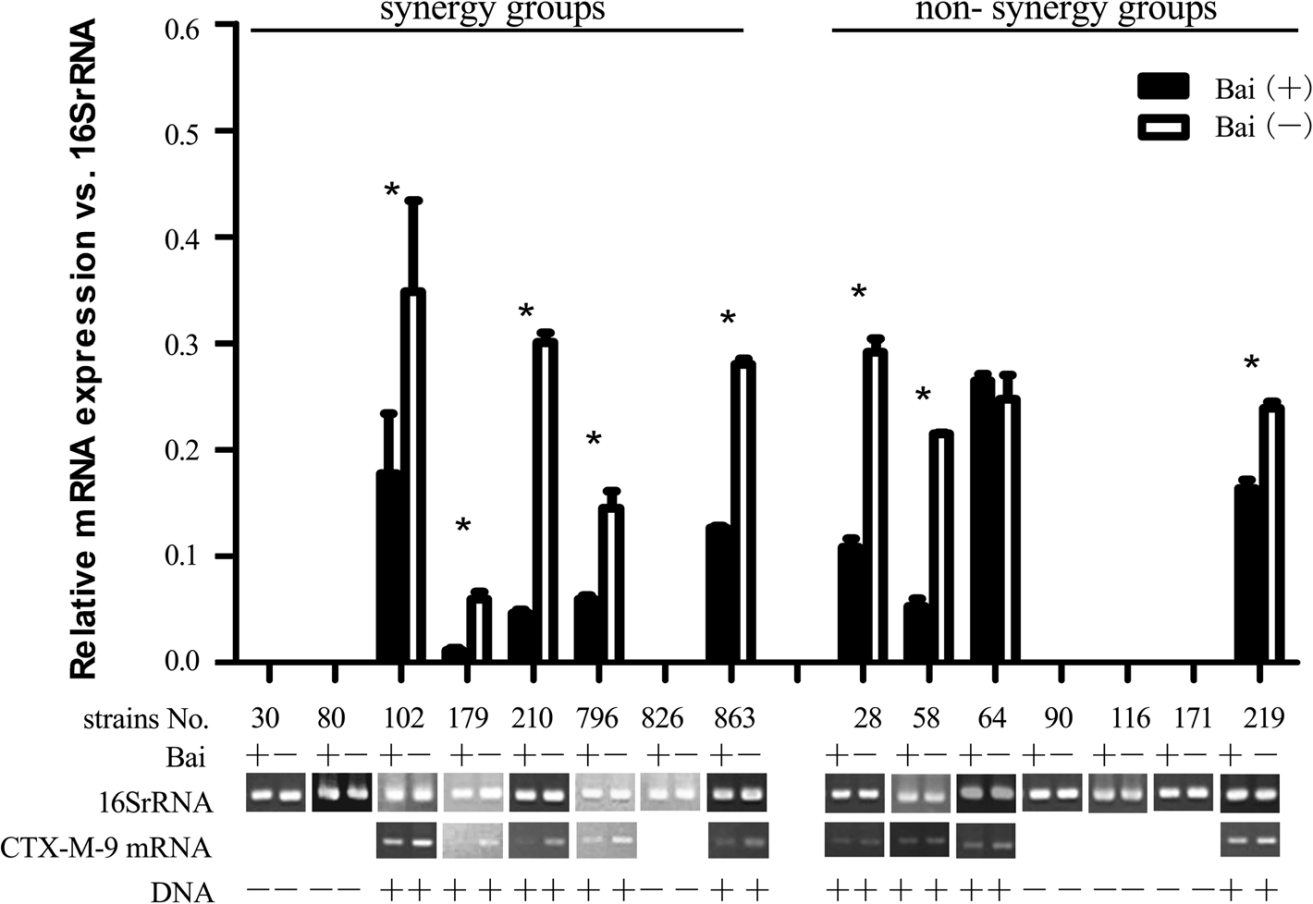
Effect of baicalein on the mRNA expression of CTX-M-9. (same as Fig. 4 in explanation)

To further investigate if baicalein interacts with cefotaxime through regulation of gene expression, 15 clinical strains of ESBL positive *K. pneumoniae* were treated with baicalein alone at the same MIC determined during synergy testing. After incubation, the effect of baicalein on mRNA expression of these resistance genes was studied using RT-PCR. The results showed that baicalein significantly inhibited the expression of CTX-M-1 in strains KP30, KP80, KP179, KP796, KP826, KP219 (*P <* 0.05) (Figs. 4, 5, and 6, Table 3). Moreover, the CTX-M-1 mRNA expression percentage inhibition (100 %, 5/5) was significantly higher than non- synergy group (25 %, 1/4) (*p* <0.05), implying that synergy of baicalein with cefotaxime may be associated with the inhibition of CTX-M-1 mRNA expression.

**Table 3:** Relationship of combined baicalein with cefotaxime and the mRNA level of resistant genes

Group	Synergy		Non- synergy		*P* value
	Inhibition(%)	Non- inhibition (%)	Inhibition(%)	Non- inhibition(%)	
TEM mRNA	100(6/6)	0(0/6)	33.3(2/6)	66.7(4/6)	0.061
CTX-M-1 mRNA	100(5/5)	0(0/5)	25(1/4)	75(3/4)	0.048*
CTX-M-9 mRNA	100(5/5)	0(0/5)	75(3/4)	25(1/4)	0.444

Based on the information in the Figs. 4, 5 and 6, the percentage of inhibited strains for each gene in synergy group and non-synergy group was compared using Fisher’s Exact Test with SPSS

*p <0.05 statistically significant

## Discussion

ESBLs play a major role in the development of antibiotic resistance in Gram- negative bacteria. It can damage the structure of β- lactam antibiotics, preventing their binding to penicillin binding protein. ESBL encoding genes consist mainly of SHV, TEM, CTX-M, OXA, GES, PER, and VEB [13]. The most common ESBL genes in *K. pneumoniae* are SHV, TEM, and CTX-M [14], among them CTX-M being the dominant gene for β- lactam antibiotic resistance in ESBL positive *K. pneumoniae* [15, 16]. Based on their amino acid changes, CTX-M type of β- lactamases are mainly divided into five groups: CTX-M-1, CTX-M-2, CTX-M-8, CTX-M-9, and CTX-M-25. CTX-M-14 (belonging to CTX-M-9 group) and CTX-M-15 (belonging to CTX-M-1 group) are two major genes in mainland China [17]. For example, a recent study identified 88 % of CTX-M-1 ESBLs among 92 CTX-M ESBL-positive strains of *K. pneumoniae* isolated from respiratory tract samples [18]. Therefore, the four commonly seen genes in mainland China, including SHV, TEM, CTX-M-1, and CTX-M-9, were selected as target resistance genes in this study.

Clavulanic acid as a commonly used β- lactamase inhibitor in practice can competitively bind with β- lactamases, forming acyl - enzyme complex to inhibit their activities, thereby cooperating with antibiotics. But clavulanic acid resistant clinical strains [19] have occurred.

Chinese herbal active ingredients, including mainly flavonoids and alkaloids, have antibacterial activity and less toxicity. Baicalein is isolated from Chinese herb as flavonoid, which has synergistic antimicrobial effects [5, 6, 20]. This study demonstrated that baicalein may cooperate with cefotaxime to inhibit ESBL positive *K. pneumoniae*. But baicalein can only partially inhibit resistant strains of ESBL positive bacteria through suppressing the mRNA expression of CTX-M-1. Meanwhile, there was no remarkable change in the number of viable bacteria when treated alone with baicalein, implying that baicalein exhibits synergistic antibacterial effect through non- bacteriostatic nor bactericidal mechanisms.

Our previous studies showed that there was difference in the mRNA expression level of ESBL resistance gene SHV in clinical strains of *K. pneumoniae*. The variation was also associated with antibiotic resistance in bacteria. Therefore we proposed a new strategy for managing bacterial resistance through regulating the expression of ESBL resistance genes [9]. However, there is no report on whether some medicine may have antibacterial effects by inhibiting the expression of resistant genes.

In this study, we first validated the synergy of baicalein with cefotaxime. Then we ruled out the possibility of bacteriostatic or bactericidal activities of synergistic baicalein. The effects of difference in resistance gene distribution on antibiotic resistance in bacterial strains were also investigated. This is the first report on interaction mechanism by which baicalein works with antibiotics through regulating the expression of resistance genes.

Relevant studies and our work showed that bacterial CTX-M gene is associated with cefotaxime resistance [11, 21]. CTX-M gene transfer experiments also confirmed that the CTX-M gene enables the bacteria to cefotaxime resistance [22]. In this study, down regulation of CTX-M-1 gene expression was found to be associated with cefotaxime MIC decrease. However, genes TEM and CTX-M-9 were not determinants of *K. pneumoniae* resistance to cefotaxime. It was shown that the gene expression of TEM, CTX-M-1, and CTX-M-9 was inhibited by baicalein in a clinical strain of bacteria, *K. pneumoniae* 219. But no synergy and cefotaxime MIC value decrease were observed. The possible reasons for this may be that this strain has various types of β- lactamase genes or other resistance mechanisms, which cover up the inhibitory effect of baicalein on the expression of certain ESBL genes.

In summary, the present study investigated the interactive effect of baicalein on bacterial drug resistance at molecular level. Our findings may pave a new way for further searching for synergistic antimicrobial drugs. More work should be done to confirm how baicalein down- regulates gene expression and why it only works in some strains.

## Conclusions

Our results demonstrated that baicalein exhibited synergistic activity when combined with cefotaxime against some of ESBL positive *K. pneumoniae* strains by inhibiting CTX-M-1 mRNA expression. However, no direct bactericidal or bacteriostatic activity was involved in the synergistic action. Baicalein seems to be a promising novel effective synergistic antimicrobial agent.

## Abbreviations

Bai, baicalein; Cla, clavulanate acid; CLSI, Clinical and Laboratory Standards Institute; DMSO, dimethyl sulfoxide; ESBL, extended- spectrum β- lactamase; ESBLs, extended- spectrum β- lactamases; FICI, fractional inhibitory concentrations index; KP, *K. pneumoniae*: *Klebsiella pneumoniae;* Mat, matrine; MHB, Mueller–Hinton Broth; MIC, minimum inhibitory concentration; MRSA, methicillin-resistant *Staphylococcus aureus*; PCR, polymerase chain reaction; RT, reverse transcription

## Additional file

Additional file 1: Table S1-1 for Fig. 2. Effects of interactive concentration baicalein on the growth of *K. pneumoniae.*(Log10(CFU/mL)). Table S1-2 for Fig. 3. Comparison of ESBL gene percentage among different groups. Table S1-3 for Fig. 4. Effect of baicalein on the mRNA expression of TEM. Table S1-4 for Fig. 5. Effect of baicalein on the mRNA expression of CTX-M-1. Table S1-5 for Fig. 6. Effect of baicalein on the mRNA expression of CTX-M-9. (XLS 22 kb)
